# Analysis of Serum and Synovial Inflammatory Markers in Periprosthetic Joint Infections: A Narrative Review

**DOI:** 10.7759/cureus.72821

**Published:** 2024-11-01

**Authors:** Amit K Yadav, Siddhartha Murhekar, Ece N Cinar

**Affiliations:** 1 Orthopaedics and Traumatology, Wrightington Hospital, Wigan, GBR; 2 Trauma and Orthopaedics, East Kent Hospitals University NHS Foundation Trust, Canterbury, GBR; 3 Trauma and Orthopaedics, St George's University Hospitals NHS Foundation Trust, London, GBR

**Keywords:** inflammation, periprosthetic joint infection, prosthetic joint infection (pji), revision, total joint arthroplasty

## Abstract

Periprosthetic joint infection (PJI) is considered a rare but devastating complication after total joint arthroplasty (TJA). The problem lies in the fact that there is a paucity of “gold standard” diagnostic tests that make the diagnosis of PJI extremely challenging. Recently, there have been increasing evidence-based guidelines that have been introduced to standardise the approach to a patient with a suspected PJI. Diagnosing a case of PJI traditionally involves initial screening for elevated serum inflammation markers C-reactive protein (CRP) (mg/dL) and erythrocyte sedimentation rate (ESR), and aspiration remains the sole confirmatory investigation. However, several factors would affect the values of the aforementioned markers, such as gender, age, and the presence of inflammatory circumstances. Serum D-dimer that detects fibrinolytic activities during infection has high sensitivity, but the specificity was not persuasive as it would elevate during other conditions, such as venous thromboembolism. Therefore, there is also a need for a simultaneous and secondary marker. There are also several synovial biomarkers, including ESR, CRP, alpha-defensin, and synovial fluid leukocyte count and differential for the detection of PJI. In this narrative review, we want to sum up the serum and inflammatory markers that have been introduced so far for detecting PJI.

## Introduction and background

Prosthetic joint infection (PJI) is a dreaded complication of total joint arthroplasty (TJA) [1]; despite being rare, it leads to surgery failure, accounting for the most common cause of revision surgery after total knee arthroplasty (TKA). Thus, PJI affects not only the patients but also the hospitals and the healthcare system. It leads to more pain, extended hospital stays, re-admission, re-operation and revision, disability, and death. The adverse health-related and economic outcomes of PJI may impose an even more significant challenge in the future, considering the aging population and growing number of arthroplasties; more than four million TJAs in a year by 2030 [2]. Clinical findings, such as fever, pain, surgical site erythema, and induration, raise suspicion for PJI, but the definite diagnosis remains challenging; nevertheless, no accurate diagnostic test is available. The microbiological testing is also limited due to the prolonged time of result production and culture-negative PJIs [3]. Many studies have used them to investigate serological, synovial, histological, and microbiological markers searching for a single or a combination of tests that would help orthopaedic surgeons in decision-making [4,5]. The widely accepted definition by the Musculoskeletal Injection Society (MSIS) for PJI suggests two positive cultures or the presence of a sinus tract are part of the major criteria being diagnostic of PJI [6]. In this article, we aim to summarize the serum and serological markers to identify the recent findings and the current gaps in PJI diagnosis by reviewing the most comprehensive related studies in PubMed and Scopus.

## Review

Synovial inflammatory markers

In case clinical and serological evaluations suggest PJI, synovial fluid analysis is the next step to make the diagnosis, even though most clinicians perform synovial aspiration simultaneous to other evaluations [7]. Synovial white blood cell (WBC) count and polymorphonuclear cell (PMN) percentage (PMN%) are included in the 2011 Musculoskeletal Infection Society (MSIS) definition of PJI [8], and, later in 2014, experts added leukocyte esterase as a minor criterion to this definition [9]. However, searching for the most accurate synovial marker for PJI is an ongoing journey, and new markers are presented constantly. In 2014, Deirmengian et al. [7] conducted a valuable study introducing several synovial markers with 100% accuracy, sensitivity, and specificity (e.g., alpha-defensin, neutrophil gelatinase-associated lipocalin (NGAL), and lactoferrin). Since then, numerous studies have implemented them, including the recent update of the PJI definition by MSIS [6].

WBC count and PMN%

Synovial WBC count and PMN% are sensitive markers for PJI, and experts recommend checking them once the synovial fluid is obtained [5]. Despite their easy and inexpensive measurement, WBC count and PMN% lack satisfactory specificities for PJI; they are affected by factors such as recent antibiotic use, dry tap, bloody aspirate, and cell counting methods. The recent update of the MSIS definition has proposed a threshold of 3,000 cells/μL for WBC count and 80 for PMN% [6] for chronic PJI, but there is still a lack of consensus, and studies have reported different values [10]. While one meta-analysis has confirmed the MSIS-suggested thresholds of WBC count [11], Tang et al. [12] suggested redefining these cut-off values because they found that a WBC count of > 4,100 cells/μL and PMN% of 70% performed better. Apart from antibiotic use, the joint location may affect the results, hence calling for joint-specific cut-off values of WBC count and PMN%.

C-reactive protein (CRP)

CRP is an inflammation marker in the blood and synovial fluid that has extensive use for infection and inflammation diagnosis; however, synovial CRP is more specific and preferred over its serological value for PJI diagnosis [13]. Since its implementation in the 2018 International Consensus Meeting (ICM) criteria for PJI, synovial CRP has been widely used in studies. However, data remain inconsistent regarding its efficacy. In the recent study of Yu et al. [14] and among 139 patients, synovial CRP with a cut-off value of 1.632 mg/L had an AUC, sensitivity, specificity, positive predictive value (PPV), and negative predictive value (NPV) of 0.861, 0.936, 0.688, 0.707, and 0.930, respectively; further analyses rejected the hypothesis that serum and synovial levels of CRP are correlated, and they suggested that, when serum CRP results are available, synovial CRP does not provide additional diagnostic value. Baker et al. [15] reviewed 588 patients; by using 2018 ICM cut-offs, they found the higher area under the curve (AUC) and specificity values for synovial CRP (AUC of 0.951 (95%CI: 0.932-0.970) with 74.2% sensitivity and 98.0% specificity. Additionally, to diagnose PJI, combined serum and synovial CRP had significantly higher AUC levels compared to serum CRP alone in their results. It needs to be clarified whether synovial CRP reflects the serum CRP diffused into the synovial fluid, but Baker et al. [15] suggest that adjunct synovial CRP is valuable for PJI diagnosis.

Alpha-defensin

Alpha-defensin, an antimicrobial peptide, primarily derives from PMNs and kills pathogens by disrupting their cell membrane. After its first use in the study of Deirmengian et al. [7], the same group discovered that the alpha-defensin immunoassay (or lateral flow) test outperforms leukocyte esterase for PJI diagnosis [16]. The enzyme-linked immunosorbent assay (ELISA) laboratory-based and lateral flow tests are the most studied methods for alpha-defensin detection, and the former is reportedly more accurate. ELISA yields an exact quantitative level of alpha-defensin but is time-consuming and expensive. The lateral flow test, on the other hand, produces fast qualitative - yes or no - results, but it is less sensitive [17]. However, according to a recent meta-analysis [18], these two methods have no significant differences in PJI diagnosis - after TKA and total hip arthroplasty (THA); they are both highly sensitive and specific. Despite their good accuracy, alpha-defensin detection methods still need further clarification. For example, the optimum dilution for the ELISA test has yet to be established; according to a study [19], 1:5000 dilution had higher sensitivity and specificity than 1:1000 dilution. Novel techniques for assessing alpha-defensin have shown excellent accuracy for PJI diagnosis, but they need to be further investigated [20]. These methods include matrix-assisted laser desorption/ionization-time of flight mass spectrometry (MALDI-TOF MS) and high-performance liquid chromatography. Apart from TKA and THA, Thiesen et al. [21] studied the effectiveness of alpha-defensin (ELISA test) in PJI diagnosis after total ankle arthroplasty. They had a sample size of 33 with the overall two diagnoses of PJI, and their results were promising. Anyhow, the accuracy of this marker in ankle and shoulder arthroplasties has yet to be studied. Despite all being said, the efficacy of alpha-defensin for PJI diagnosis is still questioned; a recent study stated that alpha-defensin has a similar proficiency to leukocyte esterase, but the latter is inexpensive and more available. Because it is highly specific for PJI, it is recommended to use alpha-defensin to confirm other biomarkers’ results [5]. Li et al. reviewed the role of alpha-defensin in the sample size of 90 PJI patients. They concluded that it did not add any evidence for PJI diagnosis, on the contrary adding $93.90 per case [22].

Leukocyte esterase 

Leukocyte esterase is an enzyme expressed in PMNs in response to various conditions and is a valuable tool for urinary tract diagnosis. It is quantitatively detected using chemical strips, allowing us to evaluate bodily fluids. Leukocyte esterase is one of the 2018 MSIS minor criteria, but data are conflicting about its efficiency and accuracy. Some studies have specifically compared leukocyte esterase to alpha-defensin in meta-analyses because they are both leukocyte-derived. In a meta-analysis of 28 studies [23], alpha-defensin and leukocyte esterase are similarly sensitive, specific, and accurate for PJI diagnosis. Li et al. [24] confirmed these findings in an updated meta-analysis of 31 studies. To address the discrepancies and conflicts, Shohat et al. [25] used the same methods to obtain preoperative leukocyte esterase and alpha-defensin lateral flow test values among 122 patients. They found that leukocyte esterase not only performs equally well as alpha-defensin but also performs better in patients with inconclusive findings. Considering the low cost and fast results of leukocyte esterase, the authors specified that alpha-defensin is not superior to it. Despite that, results are different in two recent studies investigating the performance of 2018 MSIS minor criteria. Levent et al. [26] retrospectively studied 260 TKA or THA patients; PMN% had the highest AUC in their study (0.926), followed by alpha-defensin (0.922), WBC count (0.916), and leukocyte esterase (0.861). Among an Asian population [27], an analysis of the 2018 MSIS minor criteria showed that alpha-defensin has an AUC of 0.92, while it is 0.82 for LE. It remains to be seen why these variations happen; using different measurement methods may play a role, but it needs further clarification. In the study mentioned above by Shohat et al. [25], the authors explain that clinicians should interpret leukocyte esterase based on the serological test results: when the leukocyte esterase result is concordant with an ESR and CRP, the PJI diagnosis is accurate, but, in case of discordancy, the strict thresholds of leukocyte esterase (++ or negative) should be considered [28]. The use of LE strip tests was found to lower the cost of screening for infection and shorten the time thereby saving medical resources [29].

D-lactate

Lactic acid has two isomers, L-lactate and D-lactate; the former is produced in the human body, but the latter is mainly produced and metabolized by microorganisms such as bacteria and fungi. As a result, D-lactate concentration is low in the human body and has a slow metabolism [30]; higher concentrations - especially in sterile bodily fluids such as synovial fluid - indicate infection [31]. Yermek et al. [32] studied the performance of D-lactate for diagnosing PJI -hip, knee, and shoulder. They used the working criteria of the European Bone and Joint Infection Society for PJI diagnosis. They found that D-lactate with a cut-off value of 1.263 mmol/L is 86.4% sensitive and 80.8% specific with an AUC of 90.3%. The same group tested this hypothesis in another study using institutional and MSIS criteria [33]; a cut-off of 1.3 mmol/L was highly sensitive regardless of the diagnosis criteria. Results of a recent meta-analysis also depict that D-lactate is 0.82 (95%CI: 0.70-0.89) sensitive and 0.76 (95%CI: 0.69-0.82) specific for PJI diagnosis with an AUC of 0.84 (95%CI: 0.80-0.87). Thus, studies uniformly confirm the value of this biomarker as it is an inexpensive test requiring low sample volumes and yielding fast results [34]. However, there is currently no consensus about the D-lactate cut-off value, calling for further investigations.

Interleukins (IL)

IL are inflammatory cytokines produced in response to any form of inflammation and thus have been vastly used for diagnosing immune and infectious disorders. In the early 2010s, Deirmengian et al. [35] reported that synovial IL-1 and IL-6 have an accuracy, sensitivity, and specificity of 100% for PJI diagnosis. In one study [36] and among 93 patients, researchers found that IL-1β with a 312.7 pg/mL cut-off is 97.3% sensitive and 94.6% specific for chronic PJI diagnosis. The authors further elaborate that combining synovial IL-1β and synovial PMN% is 100% specific with an NPV of 1. In another study by Su et al. [37], the synovial IL-4/IL-6 ratio, with a 382.10 pg/mL threshold, has an AUC of 0.9623, while 81.32% sensitive and 98.86% specific. ILs are also detected in serum, but their serological levels are reportedly less specific [37]. Nonetheless, combined serum and synovial IL-6 tests among 93 patients were 96.77% accurate for chronic hip and knee PJI diagnosis [38]; the cut-off values for synovial and serum levels were 1,855.36 pg/mL and 6.7 pg/mL, respectively. Low specificities are the major drawback of IL use; they increase due to various inflammatory and infection conditions, and thus their synovial level may be more accurate than their serological values. However, a recent study used synovial IL levels to differentiate between PJI and active rheumatoid arthritis [39]; they assessed IL-1β, IL-2, IL-4, IL-6, IL-8, IL-10, IL-12, and IL-17 in 102 patients and found that they underperform in this regard. Another major limitation is that there are no established cut-off values for these markers, leading to inconsistent results between studies.

Calprotectin 

Calprotectin is a 36 kDa, zinc- and calcium-binding, and cytosolic protein complex with anti-microbial activity, primarily derived from neutrophils in response to inflammation. Fecal and serum calprotectin levels are biomarkers for diagnosis and course evaluation in multiple disorders, such as inflammatory bowel disease, rheumatoid arthritis, psoriasis, and malignancies. For the first time, Wouthuyzen-Bakker et al. [40] implemented synovial calprotectin in a pilot study of 19 PJI patients and found it a valuable biomarker, especially for excluding PJI. Based on their results, a cut-off of 50 mg/L has an AUC of 94% and sensitivity, specificity, PPV, and NPV of 89%, 90%, 81%, and 95%, respectively. By the time calprotectin was introduced, more sensitive and specific biomarkers for PJI diagnosis were already approved (e.g., alpha-defensin). However, calprotectin gained enormous interest because it is accurate, cheap, and efficient. According to a recent meta-analysis [12], lab-based alpha-defensin and synovial calprotectin were the two most sensitive and specific synovial markers of PJI sensitivity of 0.91 (0.86-0.94) and 0.95 (0.88-0.98) and specificity of 0.96 (0.94-0.97) and 0.95 (0.89-0.98). Two other meta-analyses have confirmed these results. Still, as for other biomarkers, there has yet to be an agreement about the calprotectin optimum cut-off value, and the mentioned studies have used values such as 50 and 1.5 mg/mL. It also needs to be explained how calprotectin performs in case of coexisting inflammatory disorders [41,42].

NGAL

In the study of Deirmengian et al. [6], NGAL predicted PJI with 100% sensitivity and specificity for the first time. Like alpha-defensin, NGAL is an antimicrobial protein secreted from neutrophils in response to pathogens. Multiple studies have confirmed NGAL’s excellent sensitivity and specificity for PJI diagnosis [43,44]. However, the literature still needs to be improved in this regard, necessitating future cohort studies to elucidate the potential of this marker.

Serum inflammatory markers

Serum WBC is a typical test used to diagnose any infection in the body, and that is also true in prosthetic joint infections. There are many studies on WBC count in PJI with different cut-off values and variations of sensitivity and specificity. WBC's role in prosthetic joint infection diagnosis is studied by Klim et al. [45], and they found a specificity of 92%; however, a low sensitivity of 42% when using the cut-off value of 8.17 G/L. Randau et al. [46] used a value of 10,300 cells/μL for PJI and reported 95% sensitivity and a sensitivity of 21.3%. Glehr et al. decreased the WBC cut-off value to 5,480 cells/μL and achieved a good sensitivity of 91 % but at the cost of specificity of 34% [47]. Serum WBC is questionable in the diagnosis and follow-up of PJI, it is rarely elevated in chronic PJI, and its increasing value is nonspecific.

ESR and CRP 

ESR and CRP are among the most common serum markers used to seek infection because of their ease of processing and rapid results. ESR is a nonspecific inflammatory marker with variation in sensitivity from 33% to 95% and specificity from 60% to 100% [48-51]. Any Inflammation in the body leads to the release of abnormal protein from inflamed tissue, which stimulates red cell aggregation and increases the ESR. CRP is an acute inflammatory marker produced in the liver with a peak 24-35 hours after inflammation and a return to normal after two weeks. ESR and CRP are acute markers usually elevated after surgery without any source of infection. ESR and CRP are also elevated in systemic conditions such as rheumatoid arthritis, systemic lupus erythematous, trauma, and other disorders. It can vary from the laboratory; however, ESR of more than 30 mm/hour and CRP of more than 10 mg/L are considered elevated for acute prosthetic infection [8]. Almost two-thirds of culture-positive PJI have normal ESR levels, and one-third have normal CRP levels, especially in low-grade virulence organisms or patients who are already under antibiotic treatment or had previous antibiotics courses. McArthur et al. [52] found that 4% of the infected patients were negative for ESR and CRP, and the most common organism in their series was coagulase-negative staphylococcus. Infections caused by an organism of low virulence, such as propionibacterium, coagulase-negative Staphylococci, and Enterococcus faecalis, had low or normal serum CRP levels. ESR sensitivity and specificity vary from 42% to 94% and 33% to 87%, respectively, while CRP sensitivity and specificity are between 74% and 94% and 20% and 100%, respectively [53]. When ESR and CRP were both measured in parallel, their sensitivity and specificity changed to 84% and 47%, respectively [8]. An international consensus meeting in 2018 on Orthopaedic infection mentioned that ESR and CRP alone should not be relied on to rule out infection. However, serum ESR and CRP are minor criteria for periprosthetic hip and knee infection as per the 2018 definition [6]. Similarly, around 4% of people with prosthetic infections were negative for both. Therefore, serum ESR and CRP are used as the first line of screening for PJI; however, surgeons should keep in mind that negative results cannot rule out infection and should investigate with further tests if there is a possibility of infection. Shao et al. assessed the ESR, CRP, fibrinogen, and D-dimer role in the reimplantation of the prosthesis in second stage revision and the highest accuracy of serum fibrinogen, followed by CRP and lowest for D-dimer [54]. ESR is generally used as a screening tool owing to its simplicity and being cost-effective [7,55].

Neutrophil-to-lymphocyte ratio (NLR)

In bacterial infection, neutrophil count increases, and lymphocyte count decreases; thus, the NLR is helpful in wound infection. Yombi et al. [56] did a prospective study on 587 patients operated on with TKA and compared NLR to CRP level. They found that NLR came down early compared to CRP levels and potential markers to monitor after TKA. Yu et al. [57] assessed the role of NLR in 131 primary hip and knee arthroplasties with a cut-off of 2.13 and found it to be 85% sensitive and 68.3% specific for PJI. It has better accuracy than serum CRP. Ye et al. [58], with a threshold of 2.56, got a sensitivity of 57.4% and a specificity of 77.8% and found a less practical test than serum ESR and CRP. Their study of 75 prosthetic joint infections found that serum fibrinogen and CRP are better markers than NLR [59]. Festa et al. [60] did a metanalysis on NLR role in PJI, including 10 studies and a total of 2,600 patients, out of which 1,025 had PJI with a sensitivity of 72%, specificity of 74%, and AUC of 0.73. NLR is a potential research marker in prosthetic joint infection, and further research is needed to consider it a diagnostic tool.

D-dimer

**Figure 1:**
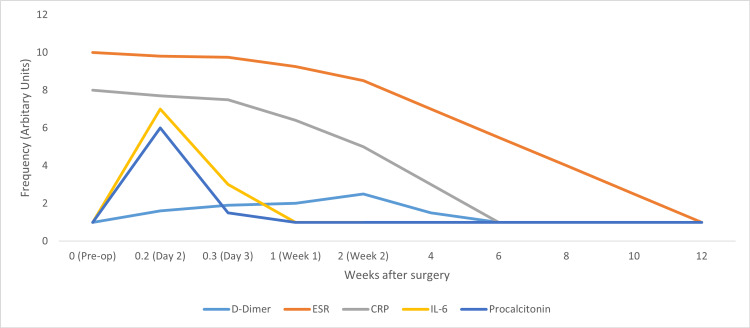
A graph depicting postoperative trends of inflammatory and infection markers: D-dimer, ESR, CRP, IL-6, and procalcitonin Sources: Refs. [54,62]

D-dimer is a degradation product of fibrin used traditionally as a screening test for the diagnosis of pulmonary embolism. It is elevated in various body infections and inflammatory pathologies such as rheumatoid arthritis. After joint replacement, D-dimer levels of more than 850 ng/mL had sensitivity and specificity of 89 and 93%, respectively, for PJI [61]. D-dimer also increased physiologically after the operation; however, it returns to average value on the second postoperative day as compared to ESR, which takes three months, and CRP around six weeks after surgery. Dimers again peak at the second postoperative week and then go back to normal at six weeks. Compared to ESR and CRP, the advantage of D-dimer is a more rapid return to normal after surgery. A graph depicting the trends of inflammatory markers is described in Figure 1 [54,62]. Sahin et al. [61] did a study on 60 patients with hip and knee arthroplasty and found that D-dimer does not help decide the time of reimplantation of prosthesis.

IL-6

IL-6 is produced by macrophages and monocytes and induces stimulation of acute phase reactant protein (e.g., CRP). Normal level is 1 Pg/mL and reaches peak level two days after surgery and then returns to normal levels. Serum IL-6 sensitivity is 49%-79%, and specificity is 58%-88% in prosthetic joint infection [46]. A meta-analysis of inflammatory markers in PJI shows IL-6 sensitivity of 97% and specificity of 91% [49]. It has also been stated that serum IL-6 levels are better than ESR, CRP, and WBC levels for detecting PJI. Serum IL-6 disadvantage is a false elevation in immunodeficiency syndrome, chronic inflammatory conditions, aseptic loosening, and cancer such as melanoma. Therefore, the combination of IL-6 and CRP better marker than alone IL-6 for low-grade infection.

Procalcitonin

Procalcitonin is a peptide precursor of calcitonin, which is released from the thyroid C cell. Procalcitonin is produced by macrophages and monocytes of the liver and is a serum marker of sepsis. The normal procalcitonin level is 0.05 ng/mL, and the half-life is 25-30 hours. It reaches a peak after six hours of sepsis [63]. Yoon et al. [64] reviewed IL-6 and procalcitonin in PJI and found that IL6 is a better marker than procalcitonin for diagnosing PJI. Procalcitonin guidance reduced antibiotic treatment for community-acquired pneumonia (CAP) from 12 to five days, with similar outcomes across all severity levels. Another multicentre trial found that PCT could also shorten antibiotic use in outpatients without affecting hospital stay, ICU admissions, quality of life, or mortality [65]. No study shows the effect of antibiotics on procalcitonin in PJI, and further research is needed. In the systematic review by Westwood et al., the role of procalcitonin-guided treatment was shown to be ≥84% cost-effective for all populations considered in the analysis [66].

TNF-α

TBF α (TNF-α) is an acute inflammatory marker also released by monocytes and macrophages. Its sensitivity is low at 43% for PJI; however, specificity is high at 94% [67]. Ettinger et al. [67] found 86% specificity and 35% sensitivity of TNF-α to differentiate between aseptic and low-grade PJI. It seems that TNF-α is not valuable for diagnosing PJI due to its low diagnostic sensitivity and long processing time needed result. The processing times for TNF-α depend on multiple factors such as assay method, and processing steps. For instance, the ELISA method takes around three to four hours (range: 3-24 hours), while rapid assays such as flow cytometry-based tests take six hours [68,69].

Fibrinogen

Fibrinogen is a fibrin precursor glycoprotein produced by hepatocytes and a part of the coagulation cycle; it stimulates the production of inflammatory cytokines such as IL-1 and TNF-α in the blood. It is also used as a predictive marker of the severity of sepsis, malaria, and appendicitis. Fibrinogen value in revision THR and TKR was studied by Klim et al. in 84 patients and compared with CRP and WBC count and had a specificity of 34% and sensitivity of 90% [45,70]. Plasma fibrinogen is one of the crucial markers useful for reimplantation prediction after prosthetic joint infection. Along with high sensitivity and specificity, fibrinogen has proven to be cost-effective in the diagnosis of PJI [45].

Platelet count and mean platelet volume

In response to infection, platelet count increases, and median platelet volume decreases, which leads to an increased ratio. Paziuk et al. [71] found a sensitivity of 48.1% and a specificity of 80.8% for platelet count and mean platelet volume for prosthetic joint infection, respectively. Their study showed specificity was better than serum ESR and CRP. Wu et al. [72] studied platelet count to mean platelet volume on 62 patients, of which 29 were a septic revision, and found it effective in diagnosing PJI.

Serum globulin and albumin-to-globulin ratio (A/G)

Globulin is a serum protein secreted by the liver and elevated in infective and inflammatory pathology, where the albumin level decreases with acute inflammation and infection. Hence, the A/G ratio decreased in infective and inflammatory conditions. Ye et al. [73] have studied the role of serum globulin and A/G ratio in 38 patients with PJI and found they can be used as diagnostic markers in PJI. Albumin, globulin, and albumin to globulin ratio are helpful in prosthetic joint infection diagnosis, and albumin is a potential marker for deciding reimplantation after infection. Wu et al. [72] also found the albumin-to-globulin ratio as a potential marker in diagnosing PJI

Intracellular adhesion molecule (ICAM)

ICAM is a glycoprotein essential for leukocyte activation, migration, and bone metabolism. Ye et al. studied its role in postoperative joint infection and found ICAM levels elevated in the infection group compared to the non-infective group. Worthington et al. [74] also found median ICAM level in septic loosening was 330 ng/mL, whereas aseptic loosening was 180 ng/mL. However, studies did not mention a cut-off value of ICAM level in PJI, and hence further research is needed.

Lipopolysaccharide binding protein (LBP)

LBP is a glycoprotein produced by the liver induced by IL-1 and IL-6 and further enhanced by TNF-α. LBP has a superior value in neonatal sepsis than other routine inflammatory markers, including procalcitonin. Friedrich et al. [75] assessed the LBP usefulness in PJI, compared it with CRP and WBC, and found a specificity of 66% and a sensitivity of 71%. The value of LBP in PJI is questionable and lower than CRP levels [53,75].

Presepsin

Presepsin is an inflammatory biomarker and soluble fraction of CD14 released from monocytes useful in grading sepsis severity. Presepsin can be used for the diagnosis and monitoring of PJI along with other inflammatory markers and is a potential candidate for further research. Serum presepsin rise after total joint arthroplasty trend is similar to CRP, and the AUC is even better than CRP. Presepsin peaks on the third day postop after arthroplasty and decreases from the fourth postoperative day [76]. Marazzi et al. [77] did a study on 30 patients of PJI to find the presepsin role in PJI and compare it with other serum biomarkers and found it is significantly elevated in the PJI group as compared to others. The summary of synovial biomarkers for PJI is presented in Table 1.

**Table 1:** Summary of periprosthetic joint infection synovial biomarkers PMN: Polymorphonuclear cells; WBC: White blood cells; LE: Leukocyte esterase; IL-6: Interleukin-6; NGAL: Neutrophil gelatinase-associated lipocalin

Biomarker	Performance	Advantages	Limitations
	Sensitivity/specificity		
PMN% and WBC count	0.88/0.89; 0.87/0.90 [9,12]	Easily calculated, low cost	Low specificity, lack of established cut-off value
LE	0.87/0.96 [21]	Inexpensive and efficient intraoperative use	Questionable performance in bloody synovial aspirate and other conditions causing increased PMN count
CRP	0.92/0.90 [14]	Not affected by metallosis	More expensive than serum CRP
Alpha-defensin	0.87/0.97 [22]	Highly specific	Expensive, questionable performance in other conditions causing increased PMN count, lack of pre-specified measurement method
D-lactate	0.82/0.76 [32]	Rapid and available, require low synovial fluid volume	Low specificity, lack of established cut-off value
IL-6	0.91/0.90 [47]	High accuracy	Costly, questionable performance in other conditions causing increased PMN count
Calprotectin	0.94/0.93 [12]	Highly accurate, available, and inexpensive	Lack of established cut-value, questionable performance in other conditions causing increased PMN count
NGAL	1 [6]	High sensitivity and specificity	Insufficient supporting data

Limitations of marker value in second-stage implantation

Assessing whether a patient is ready for reimplantation after the first stage of a staged revision arthroplasty presents a diagnostic challenge. Ghanem et al. [78] evaluated the prognostic value of ESR and CRP levels before second-stage reimplantation in two-stage revision TKA for infection. Among 109 patients, 21% required revision surgery for recurrent infection. Analysis showed that ESR and CRP needed better predictive values for detecting persistent infection, with no reliable cutoff values due to high variability. In a study of 87 hips with infected total hip arthroplasties, ESR, CRP, and synovial WBC counts were evaluated before reimplantation. While ESR and CRP often remained elevated despite infection clearance, the synovial WBC count proved the most reliable marker for detecting persistent infection, showing 78% sensitivity and 96% specificity [79]. Kusuma et al. [80] reviewed 76 patients who underwent two-stage exchange arthroplasty for infected TKA. They examined whether changes in ESR, CRP, and synovial WBC counts between stages could predict infection resolution. Despite decreases in these markers, ESR remained elevated in 54% and CRP in 21% of cases where infection had been controlled. No optimal cutoff values were found for ESR or CRP, with the synovial WBC count being the most reliable test for confirming infection control. Hoell et al. [81] evaluated the use of serum interleukin-6 (IL-6) to detect persistent infection after the first stage of a two-stage revision for PJI. In 55 patients, infection was identified in 16 cases based on positive tissue cultures. A serum IL-6 level of ≥ 13 pg/mL had a 90.9% positive predictive value for infection, while ≤ 8 pg/mL had a 92.1% NPV, indicating infection absence. IL-6 appears to be a reliable marker for assessing infection status before reimplantation. There is no ideal serum biomarker for detecting persistent infection before second-stage reimplantation. Research in this area has been limited by small sample sizes and the lack of standardized criteria for defining the success or failure of second-stage procedures. Studies from our institution suggest that the MSIS criteria, while a benchmark for diagnosing PJI, are ineffective for assessing infection resolution following explantation [82,83].

## Conclusions

ESR and CRP are first-line screening markers of PJI in infection with high-virulence organisms and patients not on any antibiotics. IL-6 and CRP combination is useful for low-grade infection. Fibrinogen and CRP are helpful in the reimplantation of the prosthesis after second-stage revision. Response of antibiotics on infection is better monitored with procalcitonin. No single serum marker can diagnose or completely exclude PJI, so combining serum marker, synovial marker, and the clinical picture is essential to analyze PJI. Nevertheless, the management of PJI requires a multi-disciplinary approach and despite established guidelines, there lies an area of little evidence needing further information.
